# Age differences in the association of prognostic nutritional index quartiles and heart failure in US adults: the NHANES 2011–2018

**DOI:** 10.3389/fnut.2025.1533632

**Published:** 2025-06-24

**Authors:** Jianlong Zhou, Yadi Li, Lv Zhu, Rensong Yue

**Affiliations:** ^1^Department of Endocrinology, Hospital of Chengdu University of Traditional Chinese Medicine, Chengdu, China; ^2^Clinical Medical School, Chengdu University of Traditional Chinese Medicine, Chengdu, China; ^3^West China Center of Excellence for Pancreatitis, Institute of Integrated Traditional Chinese and Western Medicine, West China Hospital, Sichuan University, Chengdu, China

**Keywords:** heart failure, prognostic nutritional index, NHANES, prevalence, subgroup analyses

## Abstract

**Background:**

Heart failure (HF) is the leading cause of morbidity and mortality among adults worldwide. Systemic chronic inflammatory, immune dysfunction and malnutrition are considered important characteristics of HF patients. The prognostic nutritional index (PNI) is an emerging indicator for evaluating an individual's immune-inflammatory and nutritional status. However, its relationship with the prevalence of HF is unclear. This study aimed to investigate the relationship between PNI and HF.

**Methods:**

This study included 19,965 participants from 2011 to 2018 in the National Health and Nutrition Examination Survey (NHANES) database. Weighted multiple linear regression and logistic regression, adjusted for potential confounders, were used to analyze the association between PNI and HF. Generalized additive modeling (GAM), smoothing curves, and subgroup analyses were also conducted for a deeper understanding. The diagnostic ability of the PNI for HF was assessed by analyzing the receiver operating characteristic (ROC) curve and calculating the area under the curve (AUC).

**Results:**

Unadjusted model 1 indicated a negative association between PNI and HF risk (odds ratio (OR) = 0.90, 95% CI: 0.89, 0.92), which persisted in the fully adjusted model 3 (OR = 0.97, 95% CI: 0.95, 0.99). This suggests that each unit increase in PNI reduces the likelihood of developing HF by 3%. When continuous variables were divided into quartiles, quartile 4 had a 52% lower PNI than quartile 1 (OR = 0.48, 95% CI: 0.39, 0.56). Subgroup analyses showed a significant interaction between age and the correlation between PNI and HF (interaction *P* < 0.05). Among those aged 20–59 years, the risk of developing HF was reduced by 9% for each 1-unit increase in PNI. The ROC curve showed that PNI had a high diagnostic value for HF with an AUC value of 0.642.

**Conclusions:**

The higher PNI is significantly associated with a lower prevalence of HF, particularly in the nonelderly population (20–59 years). This suggests that PNI may serve as a valuable screening tool for HF risk, emphasizing the importance of nutritional and immune status in HF development.

## 1 Introduction

Heart failure (HF) is a cluster of clinical syndromes characterized by abnormalities in the structure or function of the heart that result in high intracardiac pressures or reduced cardiac output, often accompanied by symptoms and signs such as dyspnea, fatigue, and lower-extremity edema (1, 2). HF is the leading cause of morbidity and mortality among adults globally, imposing a heavy disease burden on patients and healthcare systems each year (3). HF affects 56.19 million people worldwide in 2019, with a prevalence of 1%−3% in the global adult population and a 5-year mortality rate that remains high at 50%−75% (4, 5). In 2019, the age-standardized prevalence of HF among U.S. men is 1,291.2 per 100,000, compared with 926.2 per 100,000 for women (6). In addition, the prevalence of HF is still projected to increase in the future due to the population aging and improvements in treatment strategies and survival, although the incidence and age-standardized prevalence rates have remained stable or declined (4, 7). There is significant demographic and geographic variability in the burden of disease in HF, contributing to the multifaceted and complex nature of its healthcare spending and management (4, 6, 8, 9). Identifying modifiable risk factors and undertaking health education for physicians and patients has important public health implications (10, 11).

Although the pathogenesis of HF is still not fully understood, a systemic chronic inflammatory state/immune dysfunction and malnutrition are recognized as important features of patients with HF and are highly prevalent (12–14). Accumulating evidence suggests that several systemic inflammatory markers and abnormalities in nutritional status may predict poor prognosis in patients with HF (15, 16). Therefore, integrating chronic inflammatory response and nutritional assessment in such scenarios may help to accurately reflect populations at risk for the development and progression of HF.

The prognostic nutritional index (PNI) based on serum albumin and lymphocyte counts is an emerging indicator for assessing an individual's immune-inflammatory and nutritional status, which may theoretically represent both malabsorption and chronic inflammation in HF (17). Since PNI was first proposed to be associated with prognosis in cancer survivors, substantial subsequent clinical research has demonstrated that PNI is also strongly associated with the prognosis of other conditions, including HF (18–20). A recent meta-analysis demonstrated that being at the lowest PNI level was associated with 79% increased all-cause mortality among patients with HF (20). However, there is still a paucity of research on whether PNI is associated with the development of HF in the general population. At present, most of the clinical value of PNI is focused on the clinical prognosis of diseases. Given the accessibility and clinical relevance of PNI, the association of PNI with disease development is attracting research interest (21).

To address this research gap, we leveraged a nationally representative population-based cross-sectional study, the National Health and Nutrition Examination Survey (NHANES), to explore the association of PNI with the prevalence of HF among the general U.S. adult population. Our findings suggest that higher PNI is significantly associated with a lower prevalence of HF, suggesting that PNI may serve as a simple and accessible screening tool for HF, especially among high-risk populations.

## 2 Materials and methods

### 2.1 Study participants

The National Health and Nutrition Examination Survey (NHANES) is a comprehensive, nationwide survey aimed at assessing the health, nutrition, and sociological status of the U.S. population. Utilizing intricate, multi-stage, and probability sampling techniques, NHANES ensures representative data collection. The Mobile Examination Center (MEC) oversees the physical and laboratory examinations for this survey. All data utilized in this study are publicly accessible and anonymized, with participants providing written informed consent for health examinations at the MEC. Approval for data usage was obtained from the National Center for Health Statistics Institutional Review Board (NCHS IRB/ERB Protocol #2011-17), adhering to the Declaration of Helsinki's guidelines. Additionally, this study follows the Strengthening the Reporting of Observational Studies in Epidemiology (STROBE) guidelines to uphold reporting standards. For further study design and data details, refer to www.cdc.gov/nchs/nhanes/.

**Figure 1:**
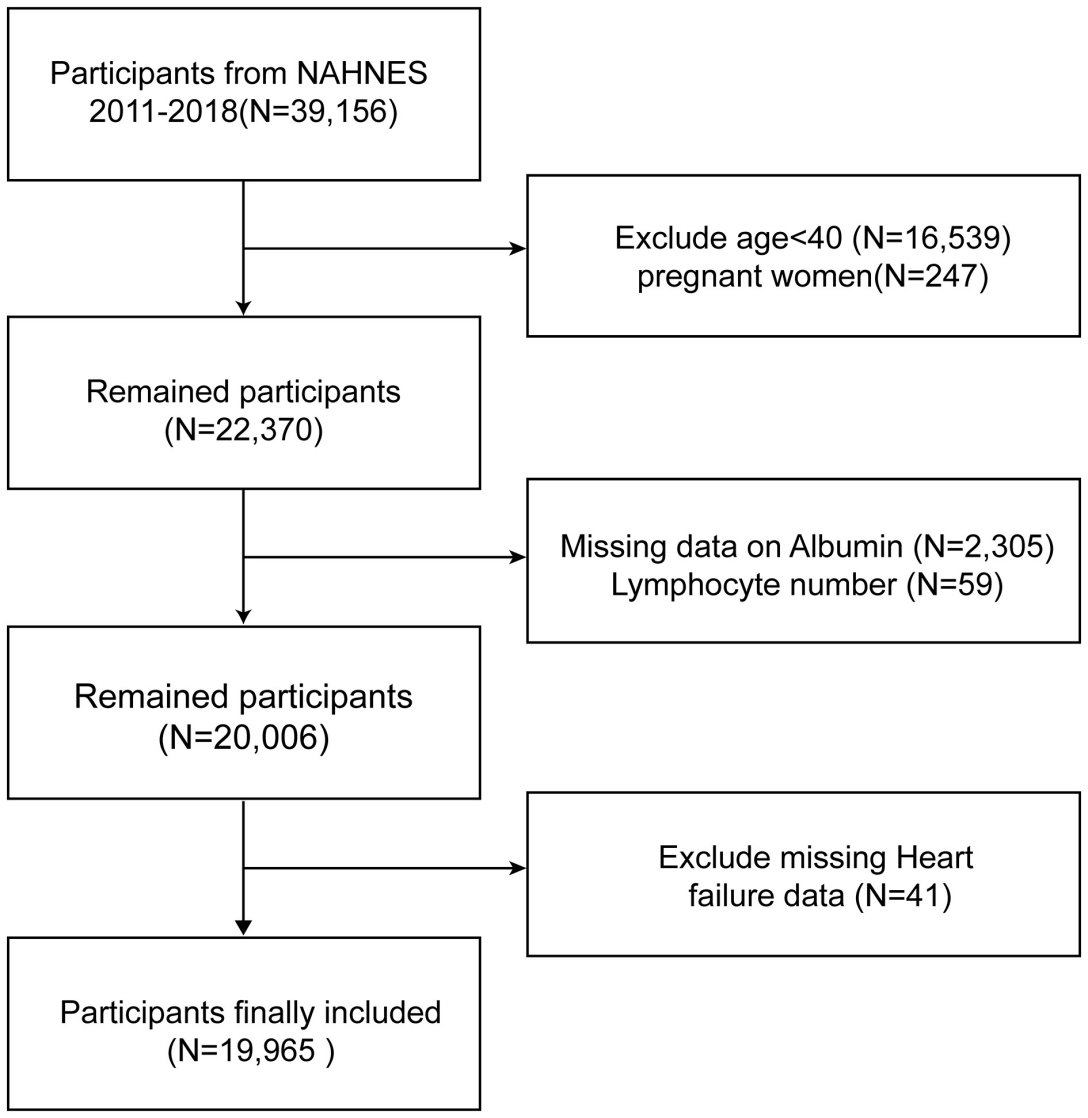
Overview of participants screening. NHANES, National Health and Nutrition Examination Survey.

Data from four 2-year cycles (2011–2018) totaling 39,156 individuals were obtained for this study, with a total of 19,965 subjects included. Figure 1 documents the full flow of the sample exclusion process. We excluded 16,539 participants who were younger than 20 years of age, 247 who were pregnant, 2305 who had missing albumin data, 59 who had lymphocyte count data, and 41 who had missing HF questionnaires, respectively.

### 2.2 Prognostic nutritional index quartiles

The Predictive Nutritional Index (PNI) evaluates individuals' nutritional status based on clinical indicators. It is calculated using the formula: PNI = 10 × serum albumin (g/dl) + 5 × lymphocyte count (10^9^/L). Lymphocyte count is primarily measured through complete blood cell (CBC) counts obtained using the Beckman Coulter method. Serum albumin levels, typically used to assess nutritional status, are measured in the NHANES database using the bromocresol purple dye method. Our analysis divides participants into PNI quartiles: Quartile 1 (Q1) reflects the lowest PNI scores, indicating a relatively higher risk of malnutrition, while Quartile 4 (Q4) reflects the highest PNI scores, indicating a relatively lower risk of malnutrition.

### 2.3 Assessment of HF

The HF test relied on responses from the MCQ questionnaire, where participants were asked if a doctor or health professional had ever diagnosed them with HF. Those who responded “yes” were categorized as having HF. This research involved secondary data analysis. Since there was no personally identifiable information, it did not necessitate institutional review.

### 2.4 Study variables

These were selected based on existing research on PNI and factors associated with HF. In this study, the selected covariates included age, gender (male or female), race/ethnicity, smoking status, diabetes, hypertension, hyperlipidemia, excessive drinking, moderate recreational activities, the ratio of family income to poverty (family PIR), education level, and body mass index (BMI). The NHANES Survey Methods and Analysis Guide offers comprehensive information regarding the methodologies employed for collecting the variables in the study. You can find more specific information on the above variables in the NHANES Methods and Analysis Guide (https://wwwn.cdc.gov/nchs/nhanes/AnalyticGuidelines).

### 2.5 Statistical analysis

All statistical analyses conducted in this study adhered to the guidelines established by the Centers for Disease Control and Prevention (CDC). PNI quartiles were subjected to *t*-tests and chi-square tests for basic participant characteristics. Linear associations between PNI and HF were analyzed using weighted multiple linear regression and logistic regression. To assess the linear association trend between PNI and HF, a trend test was conducted by transforming PNI from a continuous variable into quartiles. Additionally, generalized additive models (GAM), smoothed curve fitting, and threshold effects were employed to evaluate the potential non-linear associations between PNI and HF. In this study, three models were employed for analysis. Model 1 did not include any adjustment variables. Model 2 was adjusted for gender, age, and ethnicity. Lastly, Model 3 was adjusted for all exposure variable itself, as these factors exhibited a significant effect on the exposure factors being investigated. Subgroup analyses were conducted to explore the associations between PNI and HF among individuals of different Gender, ages, smoking status, BMI, moderate recreational activities, and hypertension status. Interaction tests were utilized to determine the consistency of these associations across the various subgroups. To assess and contrast the diagnostic performance of albumin, lymphocyte cell, and PNI for HF, we analyzed the receiver operating characteristic (ROC) curves and calculated the area under the curve (AUC). The AUC ranges from 0.5 to 1.0, with values closer to 1.0 indicating perfect predictive accuracy. Statistical analyses were carried out using R (version 4.2) and EmpowerStats (version 4.1), which are statistical computing and plotting software. A two-sided *P*-value of < 0.05 was considered statistically significant in this study.

## 3 Results

### 3.1 Characteristics of the study population

The study included a total of 19,965 participants, with 684 individuals diagnosed with HF and 19,281 without HF (Table 1). Sample size estimation was performed using precision-based calculation for a binary outcome (anticipated proportion = 3.42%, 95% CI, relative error ±20%) (22). The required minimum sample of 2,714 participants was substantially exceeded by our analytic cohort (*n* = 19,965), achieving a precision margin of 0.26% (absolute error) and ensuring adequate power for detecting clinically meaningful associations. The average age of participants with HF was significantly higher at 66.98 ± 12.36 years compared to 49.25 ± 17.42 years in those without HF (*P* < 0.001). Gender distribution showed a higher proportion of males in the CHF group (54.68%) compared to the non-HF group (48.70%; *P* = 0.002). The PNI was lower in the HF group (50.59 ± 13.93) compared to the non-HF group (53.34 ± 14.76; *P* < 0.001). Compared to the non-HF group, the HF group had lower family PIR, BMI, lymphocyte count and albumin levels, participants with a college degree or above, and participation in moderate recreational activities (*P* < 0.05). Racial and ethnic composition differed significantly, with a higher percentage of non-Hispanic Whites in the HF group (49.85%) compared to the non-HF group (37.07%; *P* < 0.001). Marital status also varied, with a higher proportion of widowed individuals in the HF group (20.91%) compared to the non-HF group (7.16%; *P* < 0.001). Smoking history revealed that 59.06% of the HF group had smoked at least 100 cigarettes in their lifetime, compared to 42.17% in the non-HF group (*P* = 0.049). Compared with the non-HF group, the HF group had a significantly higher prevalence of diabetes (43.13% vs. 13.08%), hypertension (82.46% vs. 35.51%), and hyperlipidemia (89.6% vs. 78.6%; *P* < 0.001 for all), while the proportion of individuals with excessive drinking was lower in the HF group (30.8% vs. 40.6%; *P* < 0.001).

**Table 1:** Baseline characteristics of participants between 2011 and 2018 (*n* = 19,965).

**Characteristics**	**Heart failure**		
	**No (*****N*** = **19,281)**	**Yes (*****N*** = **684)**	***P*** **-value**
Age (years)	49.25 ± 17.42	66.98 ± 12.36	< 0.001
Family PIR	2.51 ± 1.55	1.95 ± 1.29	< 0.001
BMI (kg/m^2^)	30.42 ± 7.66	29.82 ± 7.05	< 0.001
Lymphocyte number (1,000 cells/μl)	2.20 ± 2.87	2.07 ± 2.64	< 0.001
Albumin (g/L)	42.33 ± 3.45	40.25 ± 3.70	< 0.001
Gender, *n* (%)			0.002
Male	9,390 (48.70%)	374 (54.68%)	
Female	9,891 (51.30%)	310 (45.32%)	
Race/ethnicity, *n* (%)			< 0.001
Mexican American	2,700 (14.00%)	61 (8.92%)	
Other Hispanic	2,031 (10.53%)	61 (8.92%)	
Non-Hispanic White	7,147 (37.07%)	341 (49.85%)	
Non-Hispanic Black	7,147 (37.07%)	341 (49.85%)	
Other Race—Including Multi-Racial	3,207 (16.63%)	51 (7.46%)	
Marital status, *n* (%)			< 0.001
Married	9,808 (50.87%)	313 (45.76%)	
Widowed	1,381 (7.16%)	143 (20.91%)	
Divorced	2,076 (10.77%)	122 (17.84%)	
Separated	658 (3.41%)	25 (3.65%)	
Never married	3,736 (19.38%)	55 (8.04%)	
Living with partner	1,622 (8.41%)	26 (3.80%)	
Education level, *n* (%)			0.006
< 9th grade	1,805 (9.36%)	102 (14.91%)	
9–11th grade	2,368 (12.28%)	117 (17.11%)	
High school graduate or equivalent	4,261 (22.10%)	182 (26.61%)	
Some college or AA degree	5,954 (30.88%)	206 (30.12%)	
College graduate or above	4,893 (25.38%)	77 (11.26%)	
Smoked at least 100 cigarettes, *n* (%)			0.049
Yes	8,131 (42.17%)	404 (59.06%)	
No	11,150 (57.83%)	280 (40.94%)	
Diabetes status, *n* (%)			< 0.001
Yes	2,522 (13.08%)	295 (43.13%)	
No	16,245 (84.25%)	366 (53.51%)	
Borderline	514 (2.67%)	23 (3.36%)	
Hypertension status, *n* (%)			< 0.001
Yes	6,847 (35.51%)	564 (82.46%)	
No	12,434 (64.49%)	120 (17.54%)	
Hyperlipidemia status, *n* (%)			< 0.001
Yes	15,155 (78.6%)	613 (89.6%)	
No	4,126 (21.4%)	71 (10.4%)	
Excessive drinking, *n* (%)			< 0.001
Yes	7,828 (40.6%)	211 (30.8%)	
No	11,453 (59.4%)	473 (69.2%)	
Moderate recreational activities			< 0.001
Yes	8,073 (41.87%)	168 (24.56%)	
No	11,208 (58.13%)	516 (75.44%)	
PNI	53.34 ± 14.76	50.59 ± 13.93	< 0.001

Mean ± SD for continuous variables: the P-value was calculated by the weighted linear regression model. (%) for categorical variables: the P-value was calculated by the weighted chi-square test.

Family PIR, the ratio of family income to poverty; BMI, body mass index; PNI, Prognostic Nutritional Index.

Data derived from NHANES 2011–2018.

### 3.2 Association between prognostic nutritional index quartiles and the risk of heart failure

**Figure 2:**
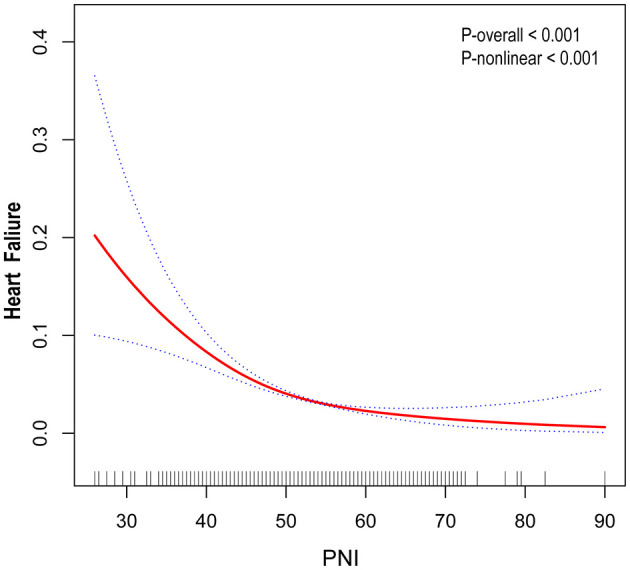
Smooth curve fitting: the relationship between PNI and heart failure.

In this study, unadjusted model 1 analysis showed a negative association between PNI and risk of HF with an odds ratio (OR) of 0.90 (95% CI: 0.89, 0.92). This association was also consistently observed in Models 2 and 3. In the fully adjusted model that accounted for all confounders (model 3), the association between PNI and risk of HF remained consistent, showing an odds ratio (OR) of 0.97 (95% CI: 0.95, 0.99). This suggests that each unit increase in PNI is associated with a 3% reduction in the likelihood of developing HF (Table 2). Outside of this, we obtained reliable results even when dividing the continuous variables into quartiles. The PNI index in quartile 4 was 52% lower than in quartile 1 (OR = 0.48, 95% CI: 0.39, 0.56; Table 2). In addition, a non-linear U-shaped correlation between PNI and HF was found by GAM and smoothing curves (*P* for non-linear < 0.001), with a steeper decline in HF risk at lower PNI levels and a plateau effect at higher values (Figure 2).

**Table 2:** Association of prognostic nutritional index with heart failure.

**Exposure**	**Model 1 (*n* = 19,965)**	**Model 2 (*n* = 19,965)**	**Model 3 (*n* = 19,965)**
	**OR (95% CI)**	**OR (95% CI)**	**OR (95% CI)**
PNI	0.90 (0.89, 0.92)	0.96 (0.95, 0.98)	0.97 (0.95, 0.99)
		< 0.001	< 0.001	< 0.001
Quartile 1	Reference	Reference	Reference
Quartile 2	0.46 (0.38, 0.56)	0.64 (0.53, 0.78)	0.68 (0.50, 0.81)
Quartile 3	0.29 (0.23, 0.37)	0.49 (0.39, 0.61)	0.50 (0.39, 0.63)
Quartile 4	0.22 (0.17, 0.28)	0.48 (0.37, 0.61)	0.48 (0.39, 0.56)
*P* for trend	< 0.0001	< 0.0001	< 0.0001

In sensitivity analysis, PNI was converted from a continuous variable to a categorical variable (quartile).

Model 1: No covariates were adjusted.

Model 2: Age, gender, and race were adjusted.

Model 3: Age, gender, race/ethnicity, diabetes status, BMI, marital status, hypertension status, hyperlipidemia status, educational level, Family PIR, smoking status, excessive drinking, and moderate recreational activities were adjusted.

OR, odds ratio; 95% CI, 95% confidence interval; Family PIR, the ratio of family income to poverty; BMI, body mass index; PNI, prognostic nutritional index.

### 3.3 Subgroup analysis

To assess the consistency of the correlation between PNI and HF across populations, we performed subgroup analyses. Subgroup analyses (Table 3) showed that the correlation between PNI and the prevalence of HF was not significantly affected by sex, BMI, smoking status, hypertension status, or activity status (*P* < 0.05). In addition, we observed a significant interaction between age and the correlation between PNI and HF (interaction *P* < 0.05). Among those aged 20–59 years, each 1-unit increase in PNI was associated with a 9% reduction in the risk of developing HF. However, the correlation between PNI and the risk of HF was not significant in those aged greater than or equal to 60 years.

**Table 3:** Results of subgroup analysis and interaction analysis.

**Subgroup**	**OR (95%CI)**	***P***	***P* for interaction**
Gender			0.487
Male	0.98 (0.94, 1.03)	0.501	
Female	0.95 (0.89, 1.03)	0.214	
Age			0.024
20–59	0.91 (0.87, 0.94)	< 0.001	
≥60	0.98 (0.92, 1.03)	0.422	
Smoking status			0.995
Yes	0.97 (0.92, 1.02)	0.276	
No	0.97 (0.90, 1.05)	0.471	
BMI (kg/m^2^)			0.929
< 30	0.97 (0.89, 1.06)	0.544	
≥30	0.97 (0.93, 1.01)	0.178	
Hypertension status			0.333
Yes	0.96 (0.92, 1.00)	0.074	
No	1.00 (0.94, 1.07)	0.995	
Moderate recreational activities			0.089
Yes	0.93 (0.90, 0.97)	0.001	
No	0.98 (0.93, 1.04)	0.511	

Age, gender, race/ethnicity, educational level, Family PIR, diabetes status, BMI, marital status, hypertension status, smoking status and moderate recreational activities were adjusted. In the subgroup analysis, the model is not adjusted for the stratification variable itself.

OR, odds ratio; 95% CI, 95% confidence interval; Family PIR, the ratio of family income to poverty; BMI, body mass index; PNI, prognostic nutritional index.

### 3.4 Predictive curve analysis

**Figure 3:**
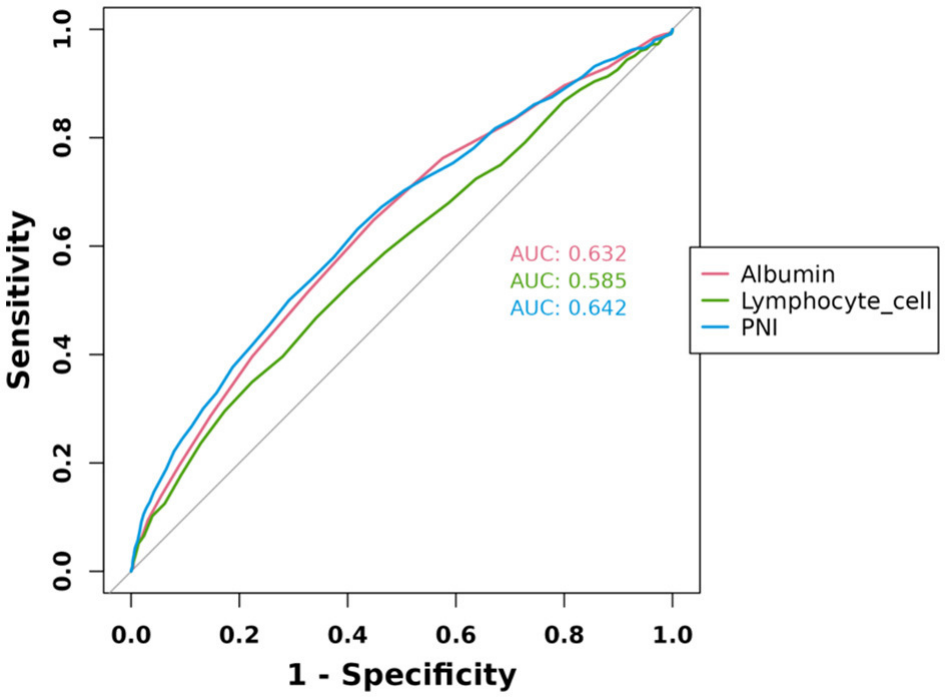
ROC curves for albumin, lymphocyte cell, and PNI prediction of HF.

ROC curve analysis of albumin, lymphocyte cell, and PNI was performed to evaluate their diagnostic ability for HF, and the results were shown in Figure 3. The results showed that the AUC of PNI was 0.642, the AUC of albumin was 0.632, and the AUC of lymphocyte cell was 0.585, which indicated that the diagnostic value of the above three indexes was in the middle range of the diagnostic ability for HF (AUC in the range of 0.5–0.7). However, the PNI had the highest AUC value among the three, indicating that it has a relatively high diagnostic value in differentiating between HF patients and non-HF patients.

## 4 Discussion

A well-validated marker for screening nutritional and immune status, the PNI, was inversely and non-linearly associated with the prevalence of HF in the general U.S. population in this large national cross-sectional analysis. Each unit increase in PNI was associated with a 3% reduction in the likelihood of HF, and those in the highest quartile of PNI had a 52% increase in the prevalence of HF compared to those in the lowest quartile. Notably, there was an age difference in this association, i.e., it was present only in the non-elderly population (20–59 years). The ROC curve also confirmed that PNI had a predictive value for HF. Overall, these findings suggest for the first time that PNI is an independent predictor of HF in the general U.S. population, particularly in the non-elderly population. Nutritional and immune status may be pathophysiologically important in the clinical development of HF and call for close attention by clinicians. PNI may be useful as a simple clinical assessment marker for screening high-risk populations for HF and for risk stratification in the general population.

The PNI is well-established in previous studies as a predictor of mortality or other adverse clinical outcomes in a variety of diseases, including several types of cardiovascular disease (CVD) (23–25). In addition, PNI has been summarized in several meta-analyses as an independent prognostic factor in patients with HF. Chen et al. (18) included 14 cohort studies showing that lower PNI was associated with increased all-cause mortality and major adverse cardiovascular outcomes (MACE) in patients with HF [hazard ratios (HR) of 1.53 and 2.26, respectively], and that each unit increase in PNI was associated with a 6% reduction in all-cause mortality and a 3% reduction in the risk of MACE in patients with HF. Another recent meta-analysis that included 12 studies demonstrated that the lowest level of PNI was associated with a significant increase in all-cause mortality and mortality plus re-hospitalization risk among HF patients compared to the highest population (HR 1.79 and 2.67, respectively), and that each unit of PNI reduction was associated with an 8% increase in all-cause mortality in patients with HF, although not with in-hospital mortality (20). However, another meta-analysis suggested that PNI may not be associated with all-cause mortality in HF with preserved ejection fraction (26). More recent clinical studies have similarly shown that PNI is significantly associated with prognosis in patients with HF (19, 27–29).

In addition to its prognostic value in diseases including HF, the association of PNI with disease development, although relatively understudied, is now gradually being recognized and attracting research interest. Recent cross-sectional analyses similarly using the NHANES have shown PNI to be significantly negatively associated with other non-communicable diseases such as diabetic kidney disease (30), migraine (31), and cognitive decline (32). Hu et al. (33) suggested that a lower PNI was associated with an increased risk of MACE among patients undergoing invasive coronary angiography (HR = 2.593). A cross-sectional analysis that included 2751 participants indicated that PNI was negatively associated with a history of CVD in a chronic kidney disease population not on dialysis (34). A cross-sectional analysis similarly using NHANES suggested that PNI in the lowest quartile was associated with a 59.3% increase in the odds of peripheral arterial disease compared with those in the highest quartile (35). However, there is still a lack of real-world research on the association of PNI with HF prevalence. Our study is the first to suggest that PNI may serve as a predictor of HF in the general U.S. population, especially in the non-elderly population. These findings provide new insights into the use of PNI as a simple and easily accessible screening tool for nutritional and immune status in the prediction and risk stratification of HF in the general population, suggesting the need to focus on populations at risk for malnutrition in high-risk populations and to deliver timely interventions accordingly.

The inverse association between PNI and HF prevalence observed in our study, particularly among non-elderly adults (20–59 years), may initially appear counterintuitive given the well-established link between overnutrition, systemic inflammation, and HF risk. However, this finding underscores the nuanced relationship between nutritional status, immune-inflammatory balance, and age-specific pathophysiology. First, PNI reflects not only nutritional sufficiency but also immune competence, integrating serum albumin (a marker of both nutritional and anti-inflammatory capacity) and lymphocyte count (an indicator of immune resilience) (36). Higher PNI likely signifies a balanced nutritional state with adequate protein reserves and effective immune regulation rather than excessive caloric intake or adiposity. This distinction is critical, as obesity-related metabolic dysfunction (e.g., insulin resistance, dyslipidemia) often coexists with malnutrition in chronic diseases, a phenomenon termed “obesity paradox” in HF populations (37). The prevalence of HF increases significantly with age and generally doubles with each additional 10 years of life, reaching more than 10% in people >75 years (1, 38). A previous large, pooled population-based cohort study demonstrated that the prospective associations of some well-recognized risk factors such as hypertension, diabetes mellitus, history of current smoking, and prior myocardial infarction with HF were more pronounced in younger populations compared to older adults (39). Our subgroup analysis revealed that the protective effect of PNI was absent in older adults (≥60 years). Although the mechanisms involved are still unknown, we speculate that the increased susceptibility to HF (40) and the possibility of having a higher burden of other chronic non-communicable diseases in the older general population partially eliminates the beneficial effects of PNI. Younger individuals, conversely, may retain greater physiological reserve, allowing PNI to better reflect protective mechanisms against early HF development. Second, systemic inflammation in HF is multifactorial (41). While obesity-driven inflammation contributes to myocardial remodeling, albumin's anti-inflammatory and endothelial-stabilizing properties, combined with lymphocyte-mediated immune surveillance, may counteract these processes in individuals with optimal PNI. Notably, our adjusted models accounted for BMI and metabolic comorbidities, suggesting that PNI's association with HF operates independently of traditional obesity-related pathways. This aligns with studies showing that malnutrition-inflammation syndrome, rather than obesity *per se*, predicts adverse outcomes in HF.

The mechanisms underlying the association of nutritional and immune status assessed by the PNI with the development of HF remain unclear. Serum albumin has physiological functions of antioxidant, anti-inflammatory, anticoagulant, antiplatelet, and maintenance of vascular permeability, all of which are important mechanisms in the development of CVD, including HF (42). Low albumin levels may permit increased vascular permeability, decreased ability to scavenge reactive oxygen species, endothelial dysfunction and decreased anticoagulant and antiplatelet capacity (33). Aberrant proportions of lymphocytes, which are important effector cells for immune response and inflammatory pathway activation, are also thought to be closely related to the pathogenic mechanisms of HF (43, 44). However, given that direct evidence is still insufficient, future studies are needed to explore the mechanisms through which PNI reduces the occurrence of HF.

This study has some significant advantages. First, it is a nationally representative, multiethnic, population-based cross-sectional analysis, making the findings potentially generalizable and replicable and providing demographic-level evidence. The association of the PNI as a simple and accessible nutritional risk screening tool with the prevalence of HF in the general U.S. population was explored for the first time with potential public health relevance. These results suggest the potential use of the PNI for screening and risk stratification for HF in the general population, especially among high-risk groups. However, our study has some important limitations. First, it was a cross-sectional analysis and thus could not derive causal associations and may have been influenced by reverse causality and residual confounders. Second, the diagnosis of HF was based on self-report in the questionnaire, which may lack accuracy and suffer from recall bias. However, previous studies have extensively validated the relevant questionnaires in NHANES and have suggested that they have good agreement (45). Subtypes, severity, and other clinical characteristics of HF were not available in NHANES, so we were unable to explore the impact of these important factors on the associations. Finally, we did not compare the value of the PNI to other nutritional screening tools for predicting HF. High-quality cohort studies are needed to further validate these findings and explore the clinical value of PNI.

## 5 Conclusion

This study reveals a significant negative association between PNI and the prevalence of HF, especially among adults aged 20–59 years. The findings emphasize the potential of PNI as a screening tool for HF risk and the importance of nutrition and immune status in the development of HF. Although the cross-sectional design limited causal inference, the consistency of results between models strengthens the evidence for a role of the PNI in HF risk stratification. Future studies should focus on elucidating the mechanisms linking PNI to HF and assessing its predictive value in different populations.

## Data Availability

The original contributions presented in the study are included in the article/supplementary material, further inquiries can be directed to the corresponding author.
